# Termination rates and histological reclassification of active surveillance patients with low- and early intermediate-risk prostate cancer: results of the PREFERE trial

**DOI:** 10.1007/s00345-020-03154-7

**Published:** 2020-03-18

**Authors:** Peter Albers, Thomas Wiegel, Heinz Schmidberger, Roswitha Bussar-Maatz, Martin Härter, Glen Kristiansen, Peter Martus, Christoph Meisner, Stefan Wellek, Klaus Grozinger, Peter Renner, Martin Burmester, Fried Schneider, Michael Stöckle

**Affiliations:** 1 Department of Urology, University Hospital Düsseldorf, Düsseldorf, Germany; 2 Department of Radiotherapy and Radiation Oncology, University Hospital Ulm, Ulm, Germany; 3 Department of Radiotherapy and Radiation Oncology, University Hospital Mainz, Mainz, Germany; 4 PREFERE Project Management, German Cancer Society, Berlin, Germany; 5 Department of Medical Psychology, University Medical Center Hamburg-Eppendorf, Hamburg, Germany; 6 Institute of Pathology, University Hospital Bonn, Bonn, Germany; 7 Department of Biometry, University Hospital Tübingen, Tübingen, Germany; 8 Institute of Medical Biostatistics, Epidemiology and Informatics, University Medical Center, Johannes Gutenberg University Mainz, Mainz, Germany; 9 Department of Urology, Klinikum Leverkusen, Leverkusen, Germany; 10 Department of Urology, Urologisches Zentrum, Lübeck, Germany; 11 Department of Urology, Vincenz Krankenhaus, Hannover, Germany; 12 Department of Urology, Klinikum Lippe, Detmold, Germany; 13 Department of Urology, University Hospital Homburg/Saar, Homburg, Germany

**Keywords:** Active surveillance, Prostate cancer, Clinical trial, Reclassification

## Abstract

**Purpose:**

Active surveillance (AS) strategies for patients with low- and early intermediate-risk prostate cancer are still not consistently defined. Within a controlled randomized trial, active surveillance was compared to other treatment options for patients with prostate cancer. Aim of this analysis was to report on termination rates of patients treated with AS including different grade groups.

**Methods:**

A randomized trial comparing radical prostatectomy, active surveillance, external beam radiotherapy and brachytherapy was performed from 2013 to 2016 and included 345 patients with low- and early intermediate-risk prostate cancer (ISUP grade groups 1 and 2). The trial was prematurely stopped due to slow accrual. A total of 130 patients were treated with active surveillance. Among them, 42 patients were diagnosed with intermediate-risk PCA. Reference pathology and AS quality control were performed throughout.

**Results:**

After a median follow-up time of 18.8 months, 73 out of the 130 patients (56%) terminated active surveillance. Of these, 56 (77%) patients were histologically reclassified at the time of rebiopsy, including 35% and 60% of the grade group 1 and 2 patients, respectively. No patients who underwent radical prostatectomy at the time of reclassification had radical prostatectomy specimens ≥ grade group 3.

**Conclusion:**

In this prospectively analyzed subcohort of patients with AS and conventional staging within a randomized trial, the 2-year histological reclassification rates were higher than those previously reported. Active surveillance may not be based on conventional staging alone, and patients with grade group 2 cancers may be recommended for active surveillance in carefully controlled trials only.

## Introduction

Prostate cancer (PCa) is the most frequent cancer in men [1]. Beginning at age 40, the likelihood of harboring prostate cancer is approximately equal to age as a percentage [2]. Approximately, half of all patients are found to have low-risk PCa with a maximum Gleason sum (GS) score of six [ISUP grade group (GG) 1]. Based on reference pathology, in a large series of patients with radical prostatectomy and GG1 cancers, the rate of lymph node metastasis was zero, and the mortality rate after 20 years was 0.2% [3, 4]. Metastasis is strongly related to the Gleason pattern and expression of metastasis-related genes, but clonal evolution from Gleason pattern 3 to Gleason pattern 4 is rare [5]. Bioptic undergrading is presumably common, implying that cancers harboring a Gleason pattern 4 component at the time of the initial biopsy can be missed by systematic biopsies. Assuming more representative sampling, patients with exclusively GG1 cancers may not need immediate treatment, and active surveillance strategies may be justified. In the pivotal randomized ProtecT trial, patients with clinically localized prostate cancers were randomized between radical prostatectomy (RP), radiotherapy (RT), and active monitoring. Based on PSA monitoring, the 10-year cancer-specific survival rate was not different [6]. In all patients managed with active monitoring (*n* = 545), eight patients died, and in the subgroup of patients with GG1 cancers (77%), only three deaths were reported after 10 years. However, these data are premature for survival analyses, as demonstrated by the SPCG-4 trial [7], which compared RP with watchful waiting and found follow-up data far beyond 20 years to still be highly relevant. Until now, the detailed inclusion criteria and monitoring schedules for active surveillance remain undefined, predominantly because of a lack of prospective randomized data. Furthermore, it remains unclear whether patients with GG2 cancers may also be eligible for active surveillance. In prospectively controlled trials, patients with GG2 prostate cancers were not necessarily more likely to experience reclassification than those with GG1 cancers. In a competing analysis, the rate of progression in patients with GG2 prostate cancers was higher than that of patients with GG1 cancer, but the cancer-specific survival rates were not significantly different [8, 9].

Based on the available data at that time, a randomized trial (PREFERE) was initiated in Germany in 2013 to prove the noninferiority of RP to RT, low-dose radiation brachytherapy (BT) and AS in patients with low- (GG1) and early intermediate-risk (GG2) prostate cancer [10]. Cancer-specific survival was defined as the primary endpoint. To facilitate patient recruitment, a preference design was used and allowed refusal of up to two trial options, as long as randomization between the remaining treatment options was accepted. Because of poor accrual, the trial was prematurely terminated in December 2016. Herein, we analyze the data of the patients randomized for and/or who chose AS in the course of this trial with a focus on the termination of AS and the histological reclassification rates with special attention to ISUP grade groups.

## Patients and methods

Patients were randomized among four possible options: RP, RT, BT, or AS. Of these four options, patients could exclude up to two choices. The final treatment was based on the randomization between the remaining treatment options. The study started patient accrual in April 2013. The design of the trial and the inclusion criteria have been described elsewhere [10]. Due to slow accrual and after sample size recalculations, the inclusion criteria were changed in 2015 to include patients with GG2 cancers (see Table 1). In addition, the use of imaging techniques (MRI, C-Trus/Anna) was allowed according to published evidence, and the inclusion of all GG1 tumors, regardless of tumor extent, was allowed [11]. To further enhance accrual, patients who had obstructive micturition disorders could be included. In December 2016, after 44 months of enrollment, the protocol was stopped for slow accrual.

**Table 1:** Inclusion and exclusion criteria of the PREFERE Trial since 2015

Inclusion criteria
Histologically verified adenocarcinoma of the prostate
At least eight biopsy cores, if modern imaging (C/Anna TRUS, MRI) is used, at least six cores were taken, and targeted biopsies only are not allowed
Treatment must start within 6 months after diagnosis
≤ cT2a
PSA ≤ 10 ng/ml
Gleason Sum Score ≤ 7a (3 + 4)
In cases of GS 7a tumors, < 33% of the cores are positive and the longest tumor area within the core < 5 mm
Men aged 18–75 with an ECOG of 0 or 1
Exclusion criteria
Contraindications for RP, RT, or BT
Surgery for BPH (TUR-P, HIFU or cryotherapy)
Life expectancy < 10 years
ASA 4
IPSS > 18^a^
Residual urine > 50 ml^a^
Prostate volume > 60 ml^a^
Predominant middle lobe BPH^a^
Proctitis, inflammatory bowel disease^a^
Use of alpha-blockers, 5-α-reductase inhibitors
Prior treatment for malignancies apart from basalioma and low-risk urothelial cancer (NMIBC)
No informed consent

^a^Patients with contraindications to radiotherapy options could be randomized as RP versus AS

The AS protocol recommended an early rebiopsy after 6 months for patients with GG2 tumors; thereafter, biopsies were recommended at 12 and 24 months. GG1 tumors had to be rebiopsied after 12 and 24 months. Thereafter, both groups had to undergo 3-years of rebiopsies until reaching 80 years of age. Clinical follow-up, including PSA values and digital rectal examination, was recommended every 3 months for the first 2 years and every 6 months thereafter.

AS was terminated if the following event occurred: (1) the patient did not wish to continue; (2) histological reclassification was observed at rebiopsy (from GG 1 to GG 2 or higher or from GG 2 to GG 3 or higher); (3) the tumor volume of GG2 tumors exceeded ≥ 33% of the biopsy cores; or (4) histological reclassification to pT3 was observed.

Aim of this analysis was to report on termination rates of the subgroup of patients treated with AS including different grade groups. The log-rank test was used to compare the time-to-reclassification between patients with GG1 and patients with GG2 tumors. Kaplan–Meier curves were produced using R, Version 3.6.

## Results

Currently, no data on the primary endpoint of cancer-specific survival are available, implying that no randomized comparisons can be made. Accordingly, the results are shown for the population of patients actually treated with AS only as exploratory analysis.

### Intention-to-treat (ITT) population

**Fig. 1:**
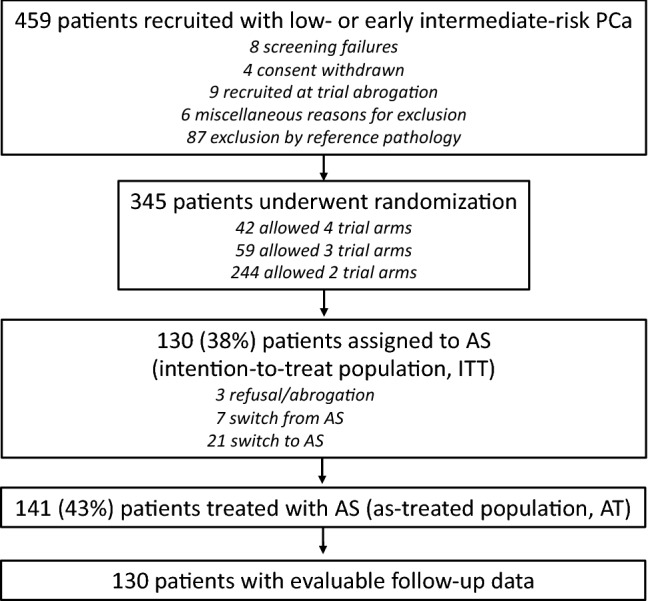
CONSORT table

Of the 345 patients randomized to the study, 130 (42%) were treated with AS (intention-to-treat population). As shown in Fig. 1, 10 patients did not accept randomization or switched to other options, and 21 patients changed from other randomization arms to the AS arm.

### As-treated (AT) population

Finally, 141 patients were treated with AS according to the trial protocol. For 130 of these patients, complete follow-up data were available with a median follow-up time of 18.8 months (range 1–48 months) (Fig. 1, Table 2).

**Table 2:** Baseline characteristics

	Total sample	Grade group 1	Grade group 2	*p* value
Gleason 7a^a^	*n* = 42 (32.3%)	–	–	–
PSA 6–10^a^	*n* = 85 (65.4%)	*n* = 55 (62.5%)	*n* = 30 (71.4%)	0.32
Number of positive biopsies^b^	2 (1–12)	2 (1–9)	2 (1–12)	0.149
Age [years]^b^	65 (48–75)	64.5 (48–75)	69 (53–75)	0.042
Size of prostate (*n* = 113)^b^	38.0 (17–71)	37.5 (17–69)	38.0 (17–71)	0.47
Erectile dysfunction (*n* = 127)^c^				0.100
Grade 0 (not present)	92 (72.4%)	66 (75.9%)	26 (65.0%)	
Grade 1	23 (18.1%)	15 (17.2%)	8 20.0%)	
Grade 2	7 (5.5%)	4 (4.6%)	3 (7.5%)	
Grade 3	5 (3.9%)	2 (2.3%)	3 (7.5%)	
Urinary incontinence (*n* = 129)^d^				1.00
Grade 0 (not present)	126 (97.7%)	85 (97.7%)	41 (97.6%)	
Grade 1	3 (2.3%)	2 (2.3%)	1 (2.4%)	

^a^Chi-Square test, ^b^Mann–Whitney test, ^c^Chi-Square test for trend, ^d^Exact test of fisher

### Termination of AS and histological reclassification

After a median follow-up time of 18.8 months, 56% (*n* = 73) of the 130 patients terminated AS.

In 56 of these 73 patients (77%), a histological reclassification, as defined above, was observed at the time of rebiopsy. Of the 56 patients with histological reclassifications, 31 (55%) and 25 (45%) had GG1 and GG2 prostate cancer at the initial biopsy, respectively. Of all 130 as-treated patients treated with AS, the GG at the first biopsy was GG1 and GG2 in 68% (*n* = 88) and 32% (*n* = 42) of the patients, respectively.

Histological reclassification occurred at a median time of 22.3 months after the first biopsy. Histological reclassification was observed in 39 (70%) and 13 (23%) patients in the first year and second or more years, respectively.

**Fig. 2:**
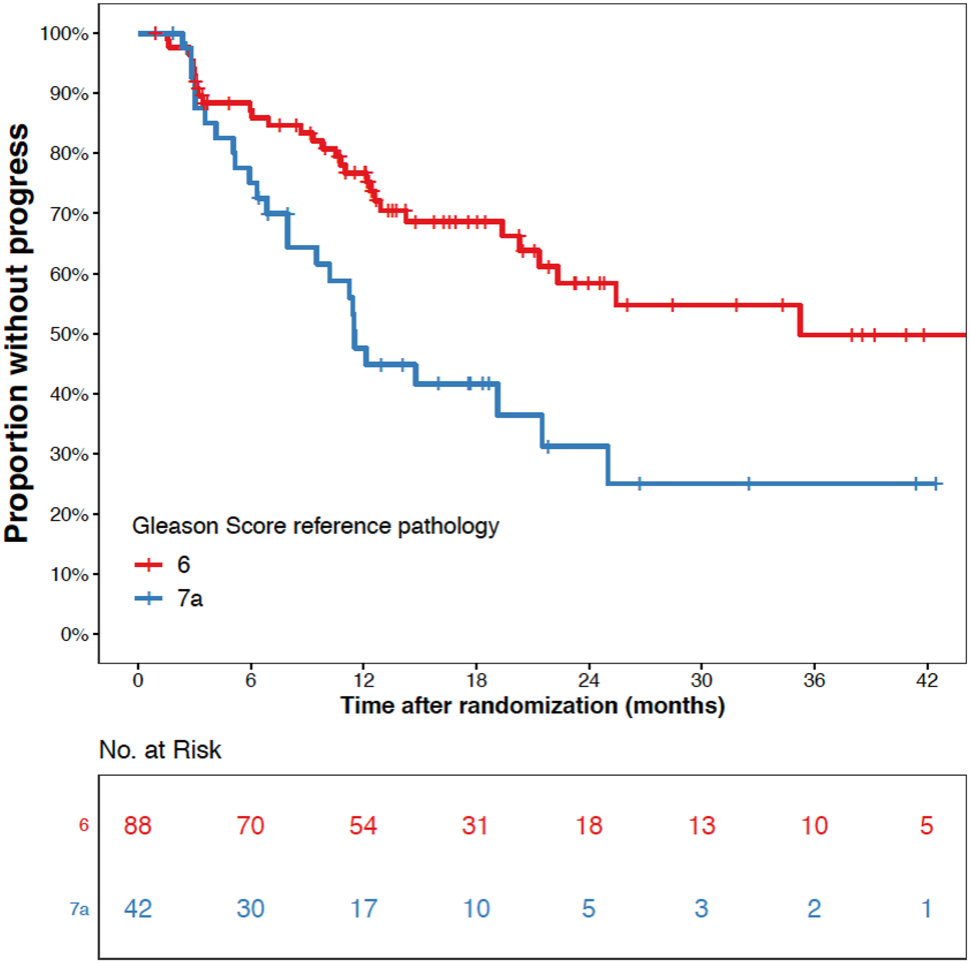
Progression-free survival of patients with active surveillance (AS) in the PREFERE trial (as-treated population) (patients with GS 6 (red) versus GS 7a (blue) tumors). Gleason 6: 31 events; Gleason 7a: 25 events

The time to histological reclassification differed significantly between patients with biopsy results of GG1 and GG2 (*p* = 0.003, see Fig. 2). At 12 months, 24% and 52% of the GG1 and GG2 patients had histological reclassification, respectively. At 24 months, 35% and 60% of the GG1 and GG2 patients had histological reclassification, respectively. A total of 37 of the 56 (66%) specimens from patients with histological reclassification were available for and confirmed by reference pathology.

AS was terminated for various other reasons in 17 patients (13%): psychological problems (*n* = 5), PSA elevation without histological confirmation of reclassification (*n* = 4), increasing voiding problems (*n* = 1), refusal to undergo rebiopsies (*n* = 3), and unknown reasons (*n* = 4).

The rebiopsy results, which were reported as the cause of AS termination, showed that 17% of the samples were GG3 and 13% were GG4 cancer.

In multivariate analysis, only ISUP grade group and pre-biopsy PSA were predictive of reclassification (see Table 3).

**Table 3:** Prognostic factor analysis for histological reclassification (univariate and multivariate analysis)

Variable	Univariate			Multivariate		
	Hazard ratio	95% CI	*p* value	Hazard ratio	95% CI	*p* value
Gleason 7a vs. 6	2.22	1.30–3.78	0.003	2.19	1.28–3.74	0.004
PSA 6–10 vs. 0–5	2.12	1.13–3.95	0.019	2.09	1.12–3.90	0.021
Number pos. biopsies	1.11	0.95–1.29	0.21	–	–	–
Age [years]	1.00	0.96–1.04	0.95	–	–	–
Size of prostate (*n* = 113)	0.99	0.97–1.02	0.56	–	–	–
Erectile dysfunction (*n* = 127)	1.08	0.77–1.51	0.66	–	–	–
Urinary incontinence (*n* = 129)	2.22	0.69–7.12	0.18	–	–	–

Note the high hazard ratio (2.22) for presence of urinary incontinence. There were only three patients with urinary incontinence, each of which showed histological reclassification. However, due to the small number of patients, the power of the related test of significance was very small

### Adherence to follow-up

The adherence to the rebiopsy plan dropped to 57% at 6 months, 60% at 12 months, and 38% at 24 months. In all, 11/141 (8%) patients had no follow-up documentation. In 15%, 8% and 8% of the patients, at least one, two, and three to nine follow-up visits were not registered, respectively.

Within 24 months, 75% of the rebiopsies were performed as requested; however, only 60% of the biopsies were performed at the requested time after the first biopsy.

### Active treatment after termination of AS

In 48 of the 73 patients (66%), active treatments after the termination of AS were reported (33 with radical prostatectomy, 11 with radiotherapy, 4 with LDR-brachytherapy).

In total, 60% and 40% of the patients who underwent radical prostatectomy (RP) after terminating AS and had histological reclassification were found to have GG1 and GG2 tumors in the radical prostatectomy specimen, respectively. No patients had radical prostatectomy specimen with tumor grades ≥ GG3.

## Discussion

The noninterventional treatment of prostate cancer, commonly termed “watchful waiting” (WW), was found to be inferior to RP with regard to long-term tumor-specific and overall survival [12]. The concept of AS aims at delaying or completely avoiding treatment through the timely detection of tumor progression. However, reliable evidence from randomized clinical trials corroborating the curative potential of AS does not exist. In particular, it is difficult to suitably select AS patients based on clinical criteria. Approximately, 20 years ago, expectant management strategies were implemented by Klotz et al. [13], tested in a consecutive cohort and finally published with a median follow-up of approximately 8 years. More than 10,000 patients have been reported to be managed with AS [2]. The only evidence-containing data on an active monitoring strategy against active treatment options are available from the ProtecT trial [6]. However, compared to the SPCG-4 trial about the WW strategy, the reported series features a follow-up of all AS patients that is too short to assess long-term efficacy. The PREFERE trial is the second multicenter trial to prospectively assess a specifically designed AS strategy in a randomized comparative trial against other options to treat prostate cancer and included patients with not only low-risk but also early intermediate-risk profiles.

The most commonly used surrogate endpoint for evaluating the success of AS strategies is the rate of termination due to histological reclassification. Precisely, this endpoint was used in the PREFERE study. The termination of AS in the PREFERE trial was not based on PSA progression alone, as is common practice in most AS strategies and in clinical routine. In addition, there were several other factors that triggered the decision of termination of AS, such as patient discomfort, increasing PSA values, psychological problems or refusal to undergo rebiopsies. In this trial, the calculated 2-year rate of switching from AS to curative treatment was twice as high as that reported in recently published large series such as the PRIAS, ProtecT and the Klotz data [6, 12, 13] (see Table 4).

**Table 4:** Histological reclassification of patients in active surveillance trials

Author	Patients	ISUP grade		Termination of AS	Follow-up (years)	Histological reclassification with repeat biopsies
		1 (%)	2 (%)			
Bul et al. [9]	2494	100		527 (21%)	1.6	415/2494 (16.6%)
Klotz et al. [13]	993	84	13	267 (27%)	6.4	40/993 (4%)
Hamdy et al. [6]	545	77		291 (54%)	10	n.a
Current date	130	67	33	73 (56%)	1.6	56/130 (40%)

In the PRIAS trial, 415 of 2494 men (16.6%) were reclassified based on repeat biopsies [14]. This may be explained by the more restrictive inclusion criteria: only patients with GG1 cancers and a maximum of two positive cores of at least eight cores from systematic biopsy were allowed. Therefore, even if early intermediate patients were excluded, the histological reclassification rate of true low-risk patients in the PREFERE trial was 35%, which is twice as high as in the PRIAS trial. This may be explained by the following points: (1) sampling error at the time of the first biopsy; (2) two, instead of only one, recommended confirmation rebiopsies within the first 2 years of the PREFERE trial; and (3) different selection criteria.

In the ProtecT trial, 23% of the 545 patients were included in active monitoring with early intermediate or intermediate and higher risk classifications (GG ≥ 3) [6]. The trigger for switching from the intervention to active treatment in the ProtecT trial was a 50% increase relative to the previous PSA value. At 2 years, the ProtecT trial found an active treatment rate in the AS population of less than 20%. This may be caused by less frequent rebiopsies compared to the PRIAS and PREFERE trials.

In the Klotz series, 993 patients had been consecutively included in an active surveillance strategy since 1995. With a mean follow-up period < 10 years, the cancer-specific survival rate was 98.5%. The inclusion criteria and follow-up strategies were comparable to those of the PREFERE trial. In comparison to the 35% of the PREFERE trials, Klotz et al. [13] reported a very low rate of true histological reclassification which finally led to termination (see Table 4). This even larger difference towards lower rates of histological reclassification in the Klotz trial compared to that in the PREFERE trial may be due to only 13% of all tumors being GG2/3 tumors, the inclusion of patients with TUR-P sampling only and a missing rebiopsy at 24 months.

From the ProtecT trial, it is known that the 10-year cancer-specific survival rate is 98.8% for patients treated with active monitoring. However, the clinical progression rate (e.g., metastasis, tumor-related obstruction) in this population at 10 years was 20.6% and a trend to an inferior survival has been already observed in an updated analysis [15]. Therefore, the cancer-specific survival data of this population compared to those of patients treated with surgery and radiotherapy will most likely decline over time [6]. In the PREFERE trial, 30% of patients had GG 3/4 histology at the time of rebiopsy, leading to discontinuation of AS. On one hand, this highlights differences in the correct evaluation of prostate histology by biopsies. On the other hand, this clearly advocates for the early detection of possibly aggressive variants at an early stage with more frequent follow-up visits, including confirmation biopsies in the first 2 years of AS. Patients in AS are always afraid of being diagnosed too late at the time of AS discontinuation. This is especially true for patients with early intermediate risk. This study suggests that frequent rebiopsies are necessary to prevent undetected high-risk GG tumors in these patients.

The overall histological reclassification rates of 35% and 60% of low-risk and early intermediate-risk patients treated with AS, respectively, still reveals problems in the correct initial stratification of prostate cancer patients for AS. Recently, data for multiparametric magnetic resonance imaging (mpMRI) at the time of inclusion in an AS strategy have gained importance [16]. Guidelines in Europe have incorporated mpMRI in the primary diagnosis of prostate cancer [17].

In addition, the high frequency of follow-up visits for future AS strategies must include mpMRIs, which are compared to an initial mpMRI at the time of inclusion in AS [18]. In the current trial, MRI was used in only three patients for initial staging, so it was not evaluable.

A prospective trial in Germany has started to assess the effect of mpMRIs substituting repeat biopsies in the first 2 years of monitoring in AS (PROMM-AS, NCT03979573).

In summary, the subgroup analysis of patients treated with AS in the PREFERE trial showed that offering AS for early intermediate-risk patients presumably entails a high risk of early histological reclassification. Even when restricted to patients with GG1 tumors, the 35% termination rate due to histological reclassification in the PREFERE trial is higher than expected. The main reasons for this difference may be suboptimal primary conventional staging and a much higher frequency of follow-up visits, including biopsies within the first 2 years, which led to histological reclassifications.

The limitations of this subgroup analysis of the PREFERE trial are the exploratory design of the analysis, the small number of patients with AS analyzed, the small number of radical prostatectomy samples for reference pathology, the early termination of the trial and the only intermediate follow-up time with lacks in follow-up data. The strengths are the data analysis of a subcohort of patients in a randomized trial, the high adherence to frequent rebiopsies and reference pathology.

## Conclusion

In summary, the subgroup analysis of patients treated with AS within the PREFERE trial showed that offering AS for patients GG1 and GG2 prostate cancer based on conventional staging entails a high risk of early reclassification. Two out of three patients with GG2 tumors terminated AS due to histological reclassification.

This observation may lead to caution in offering AS to patients with intermediate-risk cancers, and these patients may be offered AS in carefully conducted clinical trials only. AS strategies should not further rely on conventional staging but should initially include mpMRI staging.
